# Evaluation of the Effects of Surface Treatment Methods on the Properties of Coral Aggregate and Concrete

**DOI:** 10.3390/ma14226784

**Published:** 2021-11-10

**Authors:** Jinming Liu, Boyu Ju, Wei Xie, Ting Zhou, Haiying Xiao, Shanliang Dong, Wenshu Yang

**Affiliations:** 1 Defense Engineering of Academy of Military Sciences, PLA Academy of Military Sciences, Beijing 100036, China; Liujm1025@outlook.com (J.L.); xieweixiongqi@163.com (W.X.); 2 School of Materials Science and Engineering, Harbin Institute of Technology, Harbin 150001, China; 3 School of Astronautics, Harbin Institute of Technology, Harbin 150001, China

**Keywords:** coral, coral concrete, surface treatment, granulated blast furnace slag, sodium silicate

## Abstract

Coral concrete has low cost and convenient materials, making it an excellent raw material for processing. However, its lower strength limits the application of coral concrete. Surface modification is expected to increase the properties of porous coral concrete. In this study, single and compound modification treatments were applied to the surface of a coral aggregate to improve its properties for promoting the mechanical performance of coral concrete. The results showed that the micro-aggregate effect and pozzolanic activity of granulated blast furnace slag (GBFS) and the permeability and polycondensation of sodium silicate (SS) could be mutually promoted. The GBFS and SS could effectively fill the pores of the coral aggregate, enhancing the properties of the aggregate, such as density and load-bearing capacity, and reducing the water absorption and crushing index by more than 50%. GBFS and SS could intensify and accelerate the hydration of cement, and generate a large number of hard hydration products at the interfacial transition zone (ITZ), which could strengthen the bonding between the aggregate and mortar, improving the strength of the ITZ. The compressive strength of the coral concrete was significantly increased.

## 1. Introduction

Coral aggregates that are extracted from sea islands are used to fabricate concrete for island construction. The utilization of efflorescent coral aggregates can effectively save both time and expense regarding the transportation of aggregates from inland to the island. It can also successfully avoid the issues of inconvenient aggregate stacking and storage, construction constraints, and construction difficulties due to the narrow space on a sea island [1]. However, the water absorption of porous coral aggregate is high, leading to a high water–cement ratio. The high water–cement ratio and the poor strength of the coral aggregate reduce the bearing capacity of concrete. Relevant literature [1,2] states that to obtain a higher strength performance from coral concrete, the dosage of cement needs to be increased to a large extent, which increases both the cost of preparing the concrete and CO_2_ emissions.

To improve the strength of coral concrete, most researchers have been concentrating on changing the amount or type of cementitious material components. In order to improve the strength of coral concrete, researchers usually adopt methods such as adding auxiliary cementing materials, reducing the water-to-binder ratio, and adding fibers [3,4]. The method of strengthening coral aggregates improves the strength and performance of coral concrete. As a porous material, coral aggregate has basic properties, such as high porosity and high water absorption of porous materials. For porous materials, surface modification is an effective way to improve their strength [5]. Researchers used surface modification treatments to enhance porous materials, such as lightweight aggregates and recycled aggregates, to effectively improve the overall performance of concrete [6,7].

Many researchers used inorganic modification [7], organic modification [8], and biological modification [9] for the surface treatment of recycled porous aggregates for the strength enhancement of recycled concrete. Xuan et al. [10] coated the recycled aggregate from construction wastes with cement paste to improve the compressive strength and tensile strength of concrete. If the cement paste is mixed with mineral admixtures before coating the recycled aggregate, the compressive strength can also be significantly improved [11,12]. Erhan et al. [13] coated the recycled aggregate with cement and silica fume paste to prepare self-compacting concrete for promoting the utilization of recycled concrete aggregate. Nanoparticles are also often used for the surface modification of recycled aggregate [14]. Katz et al. [15] steeped the recycled aggregate with silica fume solution. The recycled aggregate was covered with a layer of silica fume particles, which greatly improved the compact degree of the ITZ, and the 28-day compressive strength was increased by 15%. Li et al. [16] used nano silica sol and nano calcium carbonate for the surface modification of recycled aggregate concrete and studied the recycled aggregate concrete from the perspective of the micro-morphology and macro-mechanical properties. Ondova and Sicakova [17] steeped the recycled aggregate with a water glass solution, which reduced the water–cement ratio of concrete and improved the flexural behavior, compressive strength, and frost resistance. PVA and silane polymer emulsion are hydrophobic. They can reduce the water absorption of porous materials [5,7]. Kou and Poon and Wan et al. [18,19] steeped recycled aggregate in PVA solution with different concentrations and studied the properties of the recycled aggregate and concrete. PVA solution helps to improve the bonding strength between aggregate and cement paste [20]. Silane polymer has a good effect on the surface modification of recycled aggregate. It can effectively reduce the water absorption of aggregate [8]. Spaeth and Tegguer [21] steeped the recycled aggregate in silane polymer solution with different concentrations and added sodium silicate to promote silane condensation for forming a thin hydrophobic membrane and reducing the hydrophilicity of the aggregate surface. Paraffin is also used for the surface treatment of porous materials [5,22,23]. On the one hand, the pores of aggregate are easily plugged after being coated with paraffin and dried. On the other hand, paraffin can react with calcium hydroxide in mortar to generate water-insoluble alkali metal salt and form a hydrophobic membrane that reduces the water absorption. In addition, researchers also used the bio-deposition method to fill the pores of porous materials so as to reduce the water absorption of porous materials and increase the strength, thereby improving the mechanical properties of concrete. Grabiec et al. [9] used sporosarcina pasteurii (*Bacillus Pasteurii*) bacteria for the surface treatment of recycled aggregate to precipitate calcium carbonate under specific conditions and improve the performance of recycled aggregate.

It was found in the review of previous research that few studies took into account the surface modification of the coral aggregate; furthermore, there is no report on the compound modification of coral aggregate. Applying a surface modification approach instead of increasing the dosage of cement can significantly reduce the cost of preparing the concrete and the CO_2_ emissions. This study mainly chose granulated blast furnace slag (GBFS) and sodium silicate (SS) as surface modifiers. The GBFS-slurry-impregnated coral aggregate can effectively physically block the pores of coral aggregate, effectively reduce the water absorption rate of coral aggregate, and improve the crushing performance of coral aggregate. At the same time, the reduction in coral aggregate water absorption can effectively reduce construction water use, reduce the water–cement ratio, and enhance the concrete strength. In addition, the GBFS slurry that is attached to the surface of the coral aggregate can chemically react with the cement hydration product, which can promote the cement hydration reaction and increase the strength of the interface between the aggregate and the cement slurry. Sodium silicate solution impregnates coral aggregates, and after solidification, it can also block the pores of coral aggregates to a certain extent and improve the basic performance of the aggregates. At the same time, the water glass that is attached to the coral aggregate can effectively promote the hydration of the cement and enhance the interface strength of the coral aggregate.

In this study, three kinds of treatment methods, namely, granulated blast furnace slag (GBFS) slurry steeping, sodium silicate (SS) impregnated, and GBFS and SS compound modification were used for the surface treatment of the coral aggregate to investigate the change rule of the crushing index and water absorption of the coral aggregate under different modification conditions. Comparisons of the concrete slump, microhardness of the ITZ, and compressive strength of the concrete with original coral aggregate and modified coral aggregate were performed. The micro-morphologies of untreated and treated coral aggregate and coral concrete were identified using SEM. The strengthening mechanisms of aggregate and concrete under different modification conditions were analyzed and compared.

## 2. Materials and Methods

### 2.1. Materials

**Figure 1:**
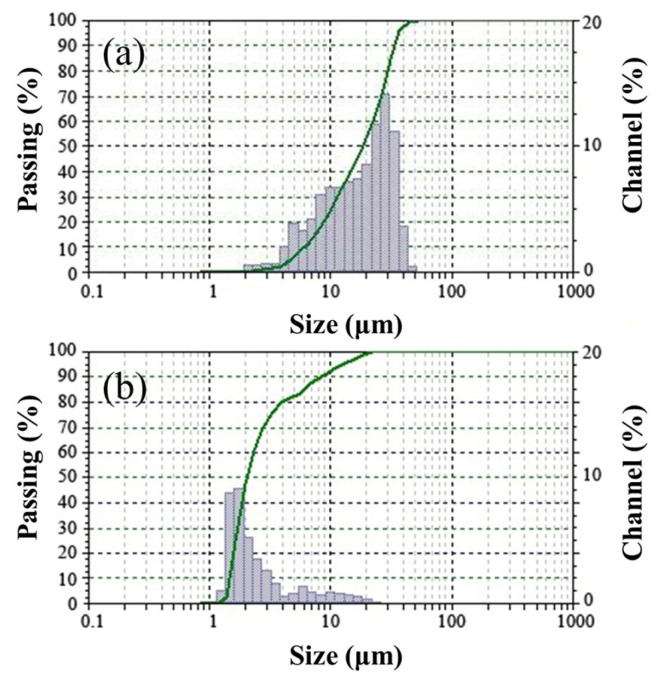
Particle size distributions of the raw materials: (**a**) cement and (**b**) blast furnace slag.

The raw materials that were used in this study were ordinary Portland cement (OPC) (P.O 42.5R), GBFS, SS, coarse coral aggregates, fine coral aggregates, and a superplasticizer. The chemical compositions of the OPC and GBFS are presented in Table 1, and their particle size distributions are provided in Figure 1. The SS was produced in Chongqing and had industrial purity, the silicon-dioxide-to-sodium-monoxide ratio of which was 3:1. The mother liquid of polycarboxylate superplasticizer, which was a thick tan liquid with a water-reducing rate of up to 30%, was adopted as the water-reducing agent.

**Figure 2:**
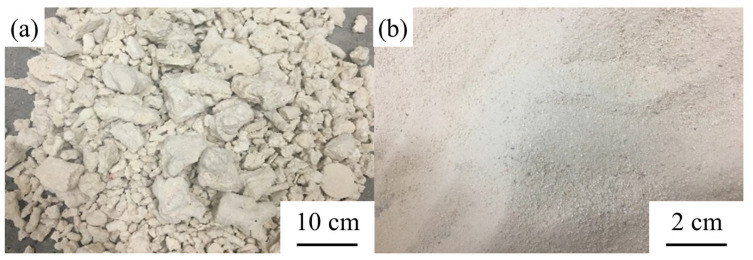
Particle morphology of the coral aggregates: (**a**) coarse coral aggregate and (**b**) fine coral aggregate.

The coral aggregates that were used in this study were collected from an oceanic island in China. The coarse coral aggregates were screened with an electric sieve shaker, which had nominal particle sizes of 5–20 mm. Table 2 exhibits the aggregate levels, which met the cumulative triage requirements in Chinese National Standard GB/T 14685-2011 “Pebble and crushed stone for construction” [24]. The aggregate percentage of particles with sizes of 9.5–16 mm was more than 50%. The macroscopic particle shape of the coarse coral aggregate is depicted in Figure 2a. The fine coral aggregate (particle size ≤ 2.36 mm) that was used in this study is pictured in Figure 2b. Table 3 lists the physical properties of the coarse coral aggregate and fine coral aggregate. The coarse coral aggregate had an irregular shape and relatively coarse surface due to the special forming process. They were porous with each having a layer of sloughed coral sand on its surface.

The concrete was prepared using the mechanical mixing method as follows: First, the cement, coarse aggregate, and fine aggregate were mixed evenly and stirred for 3 min; at the same time, the water-reducing agent and water were mixed evenly; then, the water-reducing agent mixture was added while stirring, and stirred for 2 min; finally, the mixture was stirred for 5 min to form the concrete. The rotation speed was 60–80 r/min during stirring. After the mixing was completed, it was poured into the mold, and a concrete shaker was used to form (vibration frequency was 50 ± 3 Hz) and perform standard curing after vibrating. The water temperature was 20–30 °C. After pouring into the sample, it was cured for 1 day to remove the mold and then cured for 28 days to test the performance strength of the sample.

### 2.2. Methods

#### 2.2.1. Crush Index

According to the Chinese National Standard GB/T 14685-2011 [24], 3 kg aggregates with particle sizes between 9.5 and 19 mm were poured into a round mold as required to load and unload the aggregates using a pressure testing machine. The sample in the round mold was weighed and the mass was recorded as *m*_0_; the sample was then sieved using a square screen with an aperture of 2.36 mm and the mass was recorded as *m*_1_.

The crushing indicator of the coral aggregates could be calculated using Equation (1):

(1) $$\sigma =\frac{m_{0}-m_{1}}{m_{1}}\times 100\%$$

wherein *σ* (%) represents the crushing indicator of the coarse coral aggregates, *m*_0_ (g) is the sample mass, and *m*_1_ (g) is the sample mass after crushing and screening. The arithmetic mean value of three measured values was adopted as the test result.

#### 2.2.2. Water Absorption

The coral aggregates have a strong influence on the flowability and workability of concrete. The saturated surface-dry water absorption of coarse coral aggregates was measured [24,25]. When measuring the water absorption, we ensured that the water level was 5 mm higher than the surface of the aggregates and the aggregates were completely soaked in water for 24 h. After 24 h, the water on the aggregate surface was wiped away with a wet towel to prepare the saturated surface-dry samples, and then the samples were weighed immediately. The saturated surface-dry samples were dried at the temperature of 105 ± 5 °C to a constant weight, cooled to room temperature, and weighed again.

The water absorption of the samples can be calculated using Equation (2) (accurate to 0.1%):

(2) $$W_{m}=\frac{G_{0}-G_{1}}{G_{1}}\times 100\%$$

wherein *W_m_* (%) represents the water absorption of the coarse coral aggregate, *G*_0_ (g) is the sample mass in the saturated surface-dry state, and *G*_1_ (g) is the dried sample mass. The arithmetic mean value of three measured values was adopted as the test result.

#### 2.2.3. Slump

The coral concrete slump test was performed according to the Chinese National Standard GB/T 50080-2002 [26]. Each well-mixed concrete sample was evenly filled into a slump cone layer by layer (three layers in total) and then tamped with a rod. Each layer was one-third of the cone in height after tamping. Note that the concrete should be higher than the cone verge when placing the top layer; the excessive concrete should be removed with a float to smooth out the concrete surface. After 5–10 s, the slump cone was lifted vertically and steadily, and the distance between the cone verge and the top of the slumped concrete was measured.

#### 2.2.4. Microhardness

The hardness of the interface between the aggregate and cement stone is a direct reflection of the combination of the aggregate and cement stone. An HV-1000 semi-automatic microhardness testing system was employed to measure the hardness of the interface between the coral aggregate and cement stone before and after modification. The sample was collected from the interface between the coral aggregate and cement stone in the sample concrete block after 28 days of standard maintenance. It was first soaked in absolute ethanol for 1 day to prevent hydration and cut into a 40 mm × 40 mm × 10 mm specimen. The prepared specimen was dried to a constant weight. The top and bottom surfaces of the specimen were then polished. The contact surface between the aggregate and cement stone was set as the zero-point to measure the internal aggregate and the basal body of the cement stone every 20 μm (220 μm in length). The measurement of each test point was repeated three to five times, and the mean value of the measured values was considered to be the microhardness value of the test point.

#### 2.2.5. Compressive Strength

The compressive strength test was conducted using a microcomputer-controlled electro-hydraulic servo universal testing machine according to Chinese National Standard GB/T 50081-2002 [27]. The sample was demolded after 1 day of placement and placed in a standard maintenance box for a specified period before the mechanical performance testing.

#### 2.2.6. SEM

A scanning electron microscope was employed to observe the microscopic structures of the coral aggregates and coral concrete before and after modification, especially the interface between the aggregates and mortar. The voltage was 12 kV and the working distance was about 12 mm.

#### 2.2.7. Aggregate Modification Method

For the GBFS slurry steeping, the coarse coral aggregate was steeped in 5, 10, and 20% GBFS slurry. The aggregate during the steeping process was not superimposed. The water surface was 10 mm higher than the upper layer of the aggregate. For the SS impregnation, the coarse coral aggregate was impregnated in 5, 10, and 20% SS solution. For the GBFS–SS compound modification, the aggregate was first steeped in GBFS slurry for a fixed time and then impregnated in SS solution for a certain time. The specific mixing and soaking parameters were as follows:

(1) Modification of the granulated blast furnace slag solution

The granulated blast furnace slag was mixed with water according to a certain mass percentage to form a certain concentration of granulated blast furnace slag solution. Then, the coral aggregate was added to the granulated blast furnace slag solution, stirred using mechanical stirring at a speed of 50–100 r/min for 1 h, and then left to stand for 12 h. After standing still, the coral aggregate was taken out and placed in a blast drying oven at 100 ± 5 °C to dry to a constant weight.

(2) Modification of the sodium silicate solution

A certain mass percentage of sodium silicate solution was prepared and the coral aggregate was put into the sodium silicate solution and left to stand for 5 h. After reaching the soaking time, it was removed and dried in a blast-drying box at 100 ± 5 °C to a constant weight.

(3) Compound modification

First, the coral aggregate was put into the granulated blast furnace slag solution, stirred using mechanical stirring at a speed of 50–100 r/min for 1 h, and then left to stand for 12 h. Then, the coral aggregate was taken out and placed in the sodium silicate solution to stand for 5 h. Finally, it was taken out and dried to a constant weight at 100 ± 5 °C in a blast-drying oven.

Both the GBFS and sodium silicate solutions were calculated using the mass percentage.

## 3. Results and Discussion

### 3.1. The Influence of GBFS Slurry Modification on the Performance of the Aggregate

**Figure 3:**
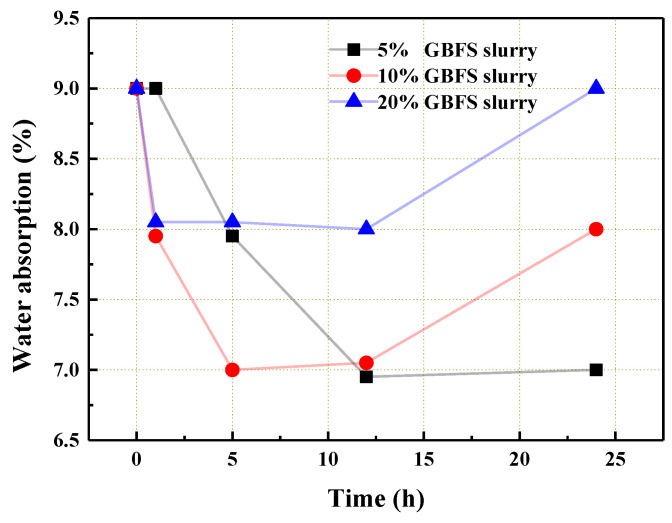
The water absorption of the coral aggregate that was treated with the GBFS slurry.

Figure 3 shows the change in water absorption of the coarse coral aggregate after being treated in a GBFS slurry. As can be seen from the figure, 5 and 10% GBFS had a better reduction effect on the water absorption of the aggregate compared to the 20% GBFS slurry. However, GBFS plugged the pores of the aggregate through physical adsorption and filling [28]. It formed no chemical barrier to prevent the entrance of water. Thus, generally, GBFS slurry had no significant effect on the reduction of water absorption of the aggregate [29].

**Figure 4:**
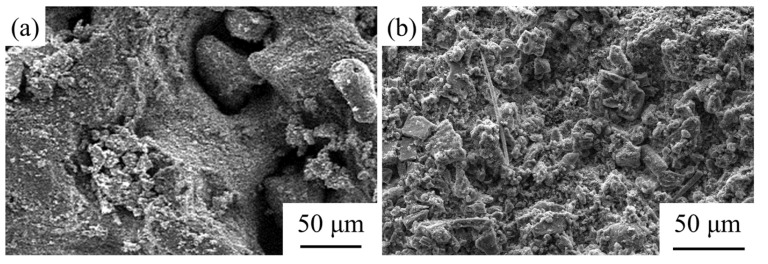
The SEM images of untreated and GBFS-treated coral aggregate: (**a**) untreated coral aggregate and (**b**) coral aggregate treated with GBFS.

Compared with the untreated coral aggregate with the polyporous and non-compacted structure pictured in Figure 4a, the aggregate that was treated with GBFS became dense and compacted, as shown in Figure 4b. Most large pores observed in Figure 4a were filled up by the GBFS slurry [30].

**Figure 5:**
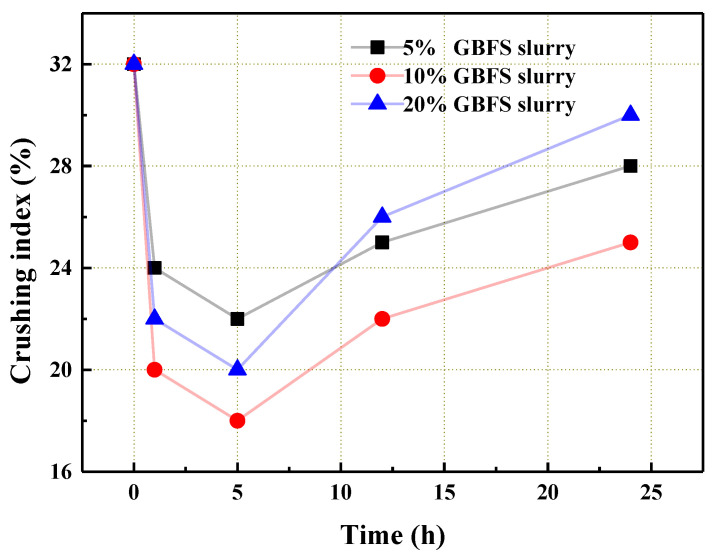
The crushing index of the coral aggregate that was treated with the GBFS slurry.

The change in the macroscopic crushing index exhibited the improvement of the aggregate’s microstructure properties. It can be seen from Figure 5 that the crushing index of the coral aggregate with GBFS modification treatment had a significant reduction. Specifically, the crushing index was at a minimum when the 10% GBFS slurry was used. Due to the micro-aggregate effect [7], GBFS could flow into the pores of aggregate and plug them, which significantly improved the load-bearing capacity of the aggregate. The crushing index with respect to the steeping time in Figure 5 showed the existence of an optimal value, above which a further increase in steeping time had a negative effect. As the immersion time was prolonged, the crushing index dropped significantly. When the content of the GBFS increased, more GBFS adhered to the surface of the aggregate, which enhanced the plugging effect. However, excess GBFS resulted in poor dispersion and fluidity of slurry, which, in turn, impaired its plugging effect. Additionally, over the optimal steeping time, with the increase in time, the crushing index increased again on account of the unstable adhesion of the excess GBFS on the surface of aggregate, i.e., the spalling of GBFS on the surface of the coral aggregate led to the increase in the crushing index. According to the above experimental results and analysis, the optimal GBFS mass fraction could be 10% or 20%, and the optimum steeping time was 5 h.

### 3.2. The Influence of SS Solution Modification on the Performance of the Aggregate

**Figure 6:**
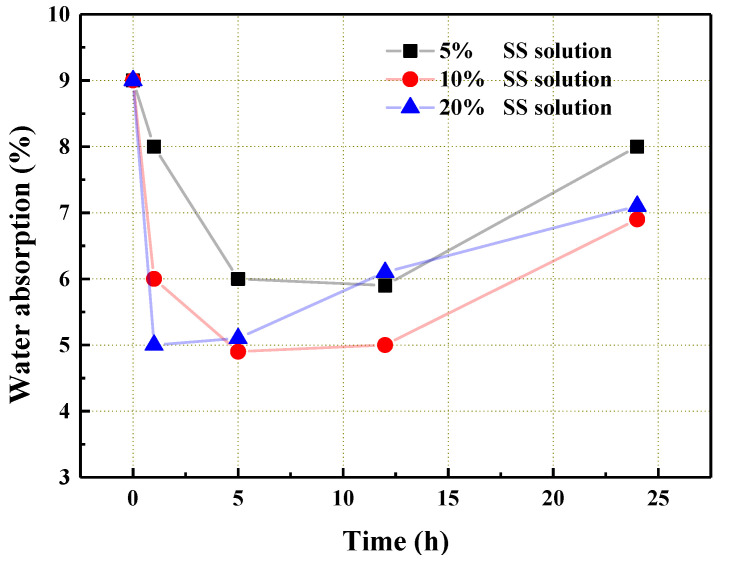
The water absorption of the coral aggregate that was treated with the SS solution.

**Figure 7:**
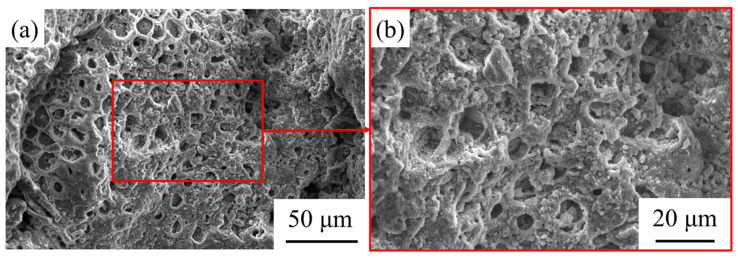
The SEM of the SS-treated coral aggregate: (**a**) morphology under low magnification and (**b**) an enlarged view of the red box area in (**a**).

Figure 6 shows the change in water absorption of the coral aggregate that was modified by the SS solution. As can be seen from the figure, SS reduced the water absorption of the aggregate by more than 40%. After the immersion, SS penetrated the open pores of the coral aggregate or adhered to the surface. During the hardening process, the silica gel was precipitated, and the small pores of the aggregate were filled [31,32]. In addition, SS on the surface of aggregate formed a layer of “adhesive film” with a reticular formation, which partially prevented moisture infiltration. The film with evenly distributed pores can be clearly observed in Figure 7. It was composed of a silica colloid, where Si-O-Si was the skeleton of the film. The formation mechanism is stated in Equations (3)–(5) [33,34]. The sodium silicate combined with water to form the colloidal silica. The film was then formed from the colloidal silicon dioxide after polycondensation and dehydration, which can be seen from Equations (3) and (4). Finally, the colloidal silicon dioxide and film together produced the net film structure, as can be seen in Equation (5).

(3) $$\begin{array}{ccc} Sodium\;silicate & & Colloidal\;silicon\;dioxide\\ M_{2}O\cdot {\mathrm{nSiO}}_{2}+(2n+1)H_{2}O & \to & 2\mathrm{MOH}+\mathrm{nSi}{\left(\mathrm{OH}\right)}_{4} \end{array}$$

(4) $$\mathrm{mSi}{\left(\mathrm{OH}\right)}_{4}\overset{Polycondensation}{\to }\underset{Polysilica\;gel}{[\mathrm{Si}{\left(\mathrm{OH}\right)}_{4}{]}_{m}}\overset{Dehydration}{\to }\underset{Film}{{\mathrm{mSiO}}_{2}}$$

(5) $$mSiO_{2}+mSi(OH{)}_{4}\to \left(\begin{array}{ccccccccccc} & | & & & & | & & & & | & \\ - & Si & - & O & - & Si & - & O & - & Si & -\\ & | & & & & | & & & & | & \\ & O & & & & O & & & & O & \\ & | & & & & | & & & & | & \\ - & Si & - & O & - & Si & - & O & - & Si & -\\ & | & & & & | & & & & | & \end{array}\right)$$

It is worth noting, however, that SS can have a hardening reaction through self-polycondensation. It is slow without external promotion. Nevertheless, SS can also be hardened by CO_2_ in the air, generating silica sol:

(6) $$Na_{2}O\cdot nSiO_{2}+\left(2n+1\right)H_{2}O+CO_{2}\to 2Na_{2}CO_{3}+nSi{\left(OH\right)}_{4}$$

**Figure 8:**
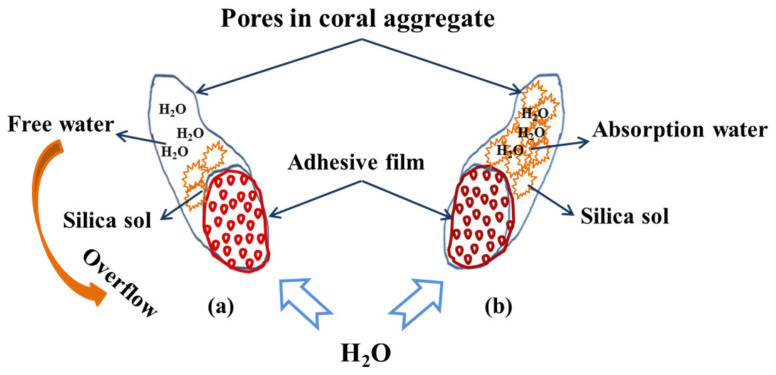
The schematic diagram of the interaction process between the SS-treated coral aggregate and water: (**a**) overflow process and (**b**) absorption process.

Moreover, the hardening reaction is also faster than the self polycondensation of SS [33,34]. On the whole, the self polycondensation and hardening reaction can account for the formation of the “adhesive film”. The insoluble silica gel, which is also called an “adhesive film”, was produced from the silica sol after dewatering and drying. The silica gel in the coral aggregate was generated from outside to inside due to the different contact times with air. The thickness of the SS adhering to the aggregate increased over the steeping time or the SS concentration, leading to a large amount of SS that persisted in the pores of the coral aggregate. The SS covering on the surface was hardened first, while the SS in the pores hardly reacted, though it still maintained good water-absorbing capacity. However, there existed a water-absorbing balance with respect to the amount of SS in the pores and the water. On the one hand, the water flowing in the pores could still overflow during the water absorption because of the small pores in the “adhesive film” (Figure 8a). On the other hand, water could be absorbed by unhardened SS and silica sol in the pores, forming absorption water (Figure 8b). The absorption water could not overflow, resulting in a high increase in water absorption over the steeping time.

Additionally, an optimal SS solution concentration and treating time were observed from the experimental results. Overall, the 10% SS solution had a better reduction effect on the water absorption of aggregate compared to the other two SS solutions used in the experiment. The optimal steeping time was 5 h.

**Figure 9:**
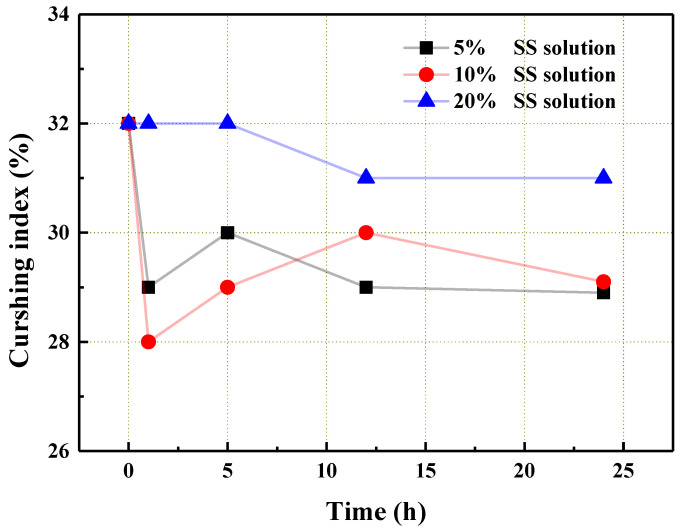
The crushing index of the coral aggregate that was treated with the SS solution.

Figure 9 illustrates the change in the crushing index with respect to the solution concentration and steeping time. As can be seen from the figure, 5 and 10% SS had a better reduction effect on the crushing index of the aggregate compared to the 20% SS solution. Furthermore, SS only physically treated the surface of aggregate. It could not react with the calcium carbonate, the main component of the coral aggregate, to produce new crystals for plugging the pores of the aggregate in the solid form [31]. Thus, generally speaking, the SS solution had no significant effect on the reduction in crushing index of the aggregate, i.e., the resistance of the aggregate to external loadings had no significant improvement. According to the above experimental analysis, the optimal SS mass fraction could be 5 or 10%, and the optimal steeping time was 5 h.

### 3.3. The Influence of GBFS–SS Compound Modification on the Performance of the Aggregate

The two single inorganic modification methods demonstrated effective respective utilities regarding improving the properties of the coral aggregate and concrete. It was notable that the GBFS treatment had a better reduction effect on the crushing index than that SS treatment. In contrast, the SS treatment had a better reduction effect on the water absorption than the GBFS treatment. In this section, based on their complementary performances, the effects of the GBFS–SS compound modification on the properties of the coral aggregate were investigated. The aggregates were first steeped in the GBFS slurry for 5 h, and then they were impregnated in the SS solution for another 5 h. Table 4 shows the experimental design for the compound modification tests, which illustrates the concentrations of the GBFS slurry and SS solution during the steeping process.

**Figure 10:**
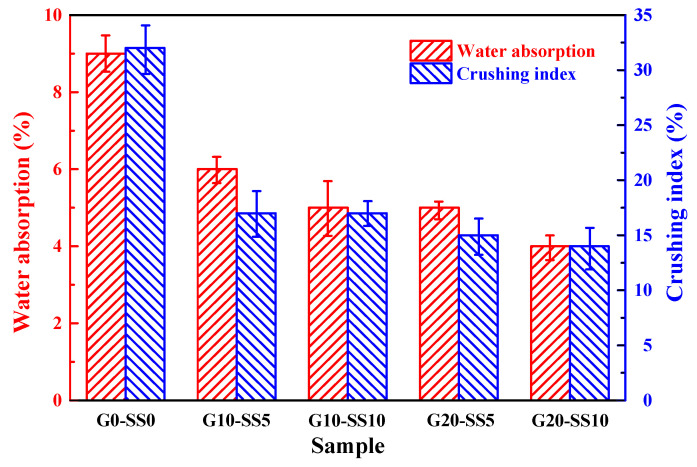
The water absorption and crushing index of the untreated and treated coral aggregates.

Figure 10 describes the water absorption and crushing index of the coral aggregate before and after modification. As can be seen from the figure, the compound modification produced more significant effects compared with the single modifications. The maximum decrease in the water absorption was 55.56% and the maximum reduction in the crushing index was 56.25%.

The substantial reduction in water absorption and crushing index of the modified aggregate was mainly due to the chemical reaction between the compound materials. The active substance of GBFS can be activated in a high-alkali environment that can be generated from an SS solution on account of its pozzolanic performance [35,36]. The glass phase is dissociated, and Ca^2+^, Si^4+^, Al^3+^, and SiO_4_^4−^ are rapidly dissolved and form a C-S-H gel after a series of polymerization reactions [37,38]. The produced gels and crystals build the initial strength of the system. They can fill the aggregate pores or attach to the aggregate surfaces, reducing the porosity and improving the strength of the aggregate [39]. During the drying process, the high temperature can accelerate the pozzolanic reaction, stimulating the activity of GBFS so as to further complete the reaction [40]. On the other hand, the Mg^2+^ in GBFS can promote the hydration process of the adhesive film of SS [7,41], where the reaction mechanism is shown in Equation (7):

(7) $$Na_{2}O\cdot nSiO_{2}+MgCl_{2}\to 2NaCl+MgSiO_{2}+\left(n-1\right)SiO_{2}$$

Thus, more and more “adhesive film” is attached to the surface of the aggregate to block water infiltration. The compound modification with GBFS slurry and SS solution had the effect of mutual promotion. The best modification condition in our present testing was first steeping the aggregate in 20% GBFS slurry for 5 h, then steeping it in 10% SS solution for another 5 h, and finally drying it to a constant weight.

Under the same mix proportion, the performances of the ordinary concrete, unmodified coral concrete, and coral concrete with different modification treatments were studied. Table 5 illustrates the changes in the concrete slump with different aggregates and modification treatments.

It can be seen from Table 5 that the slump of coral concrete was about one-third of that of the ordinary concrete under the same mix ratio and the water–cement ratio. The main reason for this was that the large porosity of coral aggregate led to higher water absorption, where some of the mixing water was absorbed in the pores of the coral aggregate, forming an internal curing effect, which reduced the amount of water that reacted with the cement. The significant reduction in reacting water facilitated the hydration of cement, leading to a great decline in the fluidity of the mixtures and a remarkable reduction in the slump of the concrete. Furthermore, the slump of coral concrete increased significantly after the coral aggregate was treated with the compound modification approach. It was shown that the main pores of the coral aggregate were plugged after the compound treatments, which caused the water absorption to be significantly reduced. Thus, less water was absorbed into the treated coral aggregate during the cement reaction. The significant reduction in water absorption of the coarse coral aggregate facilitated the hydration of cement, leading to a great improvement in the fluidity of the mixtures and the slump of coral concrete.

**Figure 11:**
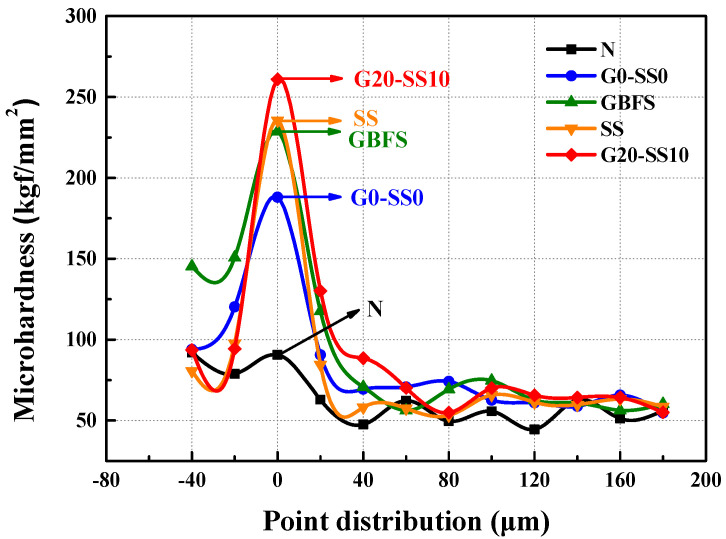
The microhardness of the ITZ of the concrete.

Hardness is an important mechanical property for the study of materials, which reflects the ability of the material to resist local plastic deformation and the ability of the tested object to resist pressure from another hard object. The microhardness test of an ITZ is an important indicator that can characterize the hardness of an ITZ of concrete and the strength of a crystal component. Figure 11 shows the testing results for concrete with different aggregates and modification treatments. GBFS stands for the coral concrete with the 20%-GBFS-slurry-coated coarse coral aggregate. SS represents the coral concrete with 10%-SS-solution-impregnated coral aggregate. The *x*-axis “0” is the position of the interface between the concrete aggregate and the cement paste, the positive axis represents the cement paste, and the negative axis is the concrete aggregate. Due to the high hardness of ordinary concrete aggregates, the microhardness was slightly higher than the interface hardness and the hardness of cement paste. However, due to the lower hardness of the coral aggregates and the lower interface hardness, the microhardness value in the negative range of the *x*-axis showed a downward trend.

It can be seen from Figure 11 that in the 0–40 μm ITZ, the microhardness of the coral aggregate concrete was significantly higher than that of the ordinary aggregate concrete, and the concrete with compound modified coral aggregate had the highest microhardness. Due to the special formation conditions of the coral aggregate, it had a relatively coarse surface structure and high water absorption. For the concrete containing the coral aggregate, the better mechanical occlusion at the interface and the more sufficient hydration of the cement led to a higher strength performance of the concrete at the interface compared with the ordinary concrete [42,43]. For the GBFS modification, a large amount of activated Al_2_O_3_ and SiO_2_ of GBFS particles were attached to the aggregate surface. They could react with the calcium hydroxide generated by the hydration of cement, which promoted the hydration process, strengthening the interface strength [44]. For the SS modification, the aggregate surface was had a large amount of attached sodium silicate, which could also react with calcium hydroxide generated via the hydration process [45,46]:

(8) $$Na_{2}O\cdot nSiO_{2}+Ca{\left(OH\right)}_{2}\to Na_{2}O\cdot \left(n-1\right)SiO_{2}+CaO\cdot SiO_{2}+H_{2}O$$

(9) $$3CaO\cdot Al_{2}O_{3}\cdot nH2O+3Na_{2}O\cdot SiO_{2}\to 3CaO\cdot SiO_{2}\cdot nH_{2}O+3Na_{2}O\cdot Al_{2}O_{3}$$

It promoted the hydration of cement and produced a hydraulic calcium silicate colloid, which improved the mechanical occlusion of the coral aggregate and cement, enhancing the interface strength [47]. For the GBFS–SS compound modification, the significant enhancement in microhardness resulted from the combined effects, which could facilitate the chemical reactions of the GBFS and SS, and promote the hydration process, thus generating more adhesive materials and hard crystals, and increasing the cohesion between the aggregate and the cement paste [48]. In the 40–180 μm ITZ, the strength of the mortar played a dominant role with the same mix proportion.

**Figure 12:**
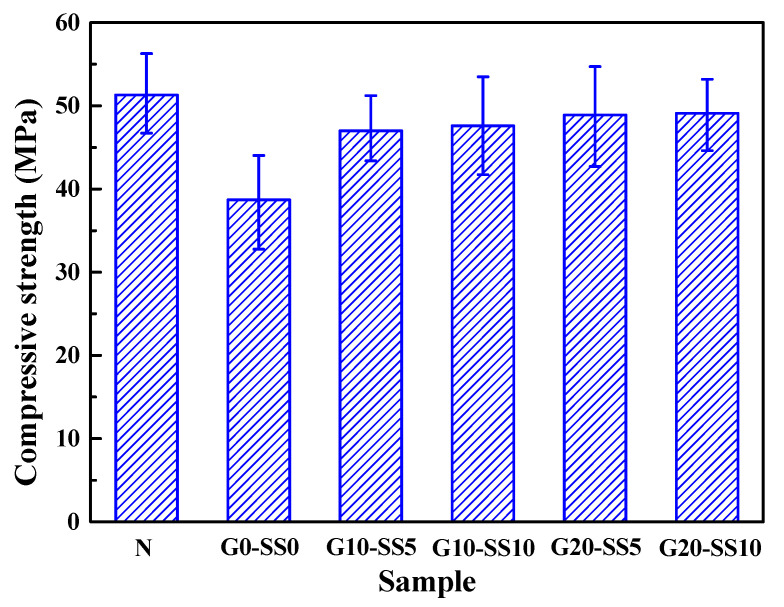
The compressive strengths of the concrete samples.

Figure 12 shows the compressive strengths of the concrete with different aggregates and modification treatments. As can be seen from the figure, although the ordinary concrete had the highest compressive strength, the strength of the coral concrete with the compound modification treatment was only 2.2 MPa lower than that of the ordinary concrete. Additionally, the compressive strengths of the treated coral concrete were all higher than that of the untreated coral concrete. Such an increase in the strength of the concrete was mainly due to the increase in the strength of the aggregate and the interface strength. The crushing index and interface strength of the coral aggregate with an effective modification treatment were much stronger than that of the unmodified coral aggregate [49,50].

**Figure 13:**
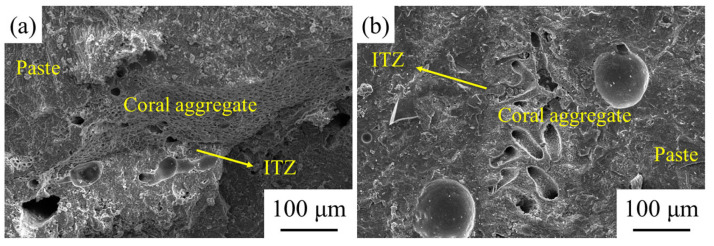
The SEM images of untreated and treated coral concrete: (**a**) ordinary coral concrete and (**b**) coral concrete with modified aggregate.

The micro-morphologies of the unmodified coral concrete and coral concrete with compound modification (in the A4 condition) are depicted in Figure 13. The incompact bonding between the untreated coral aggregate and mortar is observed in Figure 13a [51,52]. By contrast, the coral aggregate could bond with the mortar compactly through the compound modification treatment. There are no obvious gaps shown in Figure 13b. The SEM images indicated that the compound modification had a positive impact on improving the occlusion performance between the aggregate and mortar [52,53].

## 4. Conclusions

In this study, GBFS slurry, SS solution modification, and GBFS–SS compound modifications were used for the surface treatment of a coral aggregate to study the changes in properties of the coral aggregate and coral concrete. The results indicated that GBFS slurry had no significant reduction effect on the water absorption of the aggregate, and it could significantly reduce the crushing index. In contrast, the SS solution had significantly reduced the water absorption, and it had no significant impact on the reduction of the crushing index. There existed an optimal steeping time, above which a further increase in time had a negative effect. Through the GBFS–SS compound modification method, the water absorption and crushing index of the coral aggregate were greatly reduced. The water absorption and crushing index were decreased by 55.56 and 56.25%, respectively. The slump of coral concrete with the GBFS–SS modification treatment was increased on account of the effective filling of the pores of the aggregate. The compressive strength of such concrete was 26.88% higher than that of the untreated coral concrete and was only 2.2 MPa lower than that of ordinary concrete under the same mix proportion. The reason for this phenomenon was that the SS stimulated the active substance in GBFS to depolymerize and polymerize to form a C-S-H gel, and metal ions, such as Mg^2+^, in the GBFS promoted SS polycondensation to form a film. The two promoted each other and significantly enhanced the filling effect of the aggregate pores.

## Figures and Tables

**Table 1:** Chemical properties (wt.%) and physical properties of the experiment materials.

Properties	CaO	SiO_2_	Al_2_O_3_	Fe_2_O_3_	MgO	Na_2_O	MnO	K_2_O	SO_3_	Loss on Ignition	Powder Density (g/cm^3^)	Specific Density (g/cm^3^)	Specific Surface Area (m^2^/kg)
OPC	64.13	21.43	2.24	3.78	2.07	0.78	-	-	2.25	3.32	1.4–1.6	3.0–3.1	350–400
GBFS	45.75	31.4	12.3	0.79	5.25	0.42	0.51	0.37	2.32	0.89	1.0–1.3	2.8–3.0	400–450

**Table 2:** Accumulated retained percentage of coarse coral aggregate.

Bore diameter (mm)	26.5	19	16	9.5	4.75	2.36
The cumulative triage (%)	0	8	20.5	77	92.8	97.9

**Table 3:** Basic properties of the coarse coral aggregate and fine coral aggregate.

Basic Properties	Packing Density (kg/m^3^)	Close Packing Density (kg/m^3^)	Bibulous Rate (%)	Silt Content (%)	Chloride Ion Content (%)
Coarse coral aggregate	1264	1380	9	2.35	0.074
Fine coral aggregate	1115	1225	5	0.50	0.052

**Table 4:** Compound modification tests for the coral aggregate.

Number	GBFS (%)	SS (%)
G0-SS0	0	0
G10-SS5	10	5
G10-SS10	10	10
G20-SS5	20	5
G20-SS10	20	10
GBFS	20	0
SS	0	20

**Table 5:** The slumps of concrete with different aggregates and modification treatments.

Specimen	Cement (kg/m^3^)	Aggregate (kg/m^3^)	Sand Rate	Water (kg/m^3^)	Superplasticizer (kg/m^3^)	Slump (mm)
N	500	750	36%	200	2	93
G0-SS0	500	750	36%	200	2	36
G10-SS5	500	750	36%	200	2	47
G10-SS10	500	750	36%	200	2	68
G20-SS5	500	750	36%	200	2	43
G20-SS10	500	750	36%	200	2	72
GBFS	500	750	36%	200	2	38
SS	500	750	36%	200	2	42

N stands for ordinary concrete, the particle size was 5–19 mm, the sand was ordinary river sand, and the fineness modulus was 2.6; A0 represents untreated coral concrete.

## Data Availability

The data that is presented in this study are available upon request from the corresponding author.
